# Pre-encoding gamma-band activity during auditory working memory

**DOI:** 10.1038/srep42599

**Published:** 2017-02-15

**Authors:** Jochen Kaiser, Maria Rieder, Cornelius Abel, Benjamin Peters, Christoph Bledowski

**Affiliations:** 1 Institute of Medical Psychology, Medical Faculty, Goethe University, Frankfurt am Main, Germany; 2 Max-Planck Institute for Empirical Aesthetics, Frankfurt am Main, Germany

## Abstract

Previous magnetoencephalography (MEG) studies have revealed gamma-band activity at sensors over parietal and fronto-temporal cortex during the delay phase of auditory spatial and non-spatial match-to-sample tasks, respectively. While this activity was interpreted as reflecting the memory maintenance of sound features, we noted that task-related activation differences might have been present already prior to the onset of the sample stimulus. The present study focused on the interval between a visual cue indicating which sound feature was to be memorized (lateralization or pitch) and sample sound presentation to test for task-related activation differences preceding stimulus encoding. MEG spectral activity was analyzed with cluster randomization tests (N = 15). Whereas there were no differences in frequencies below 40 Hz, gamma-band spectral amplitude (about 50–65 and 90–100 Hz) was higher for the lateralization than the pitch task. This activity was localized at right posterior and central sensors and present for several hundred ms after task cue offset. Activity at 50–65 Hz was also increased throughout the delay phase for the lateralization compared with the pitch task. Apparently cortical networks related to auditory spatial processing were activated after participants had been informed about the task.

Working memory is related to the interplay of attention control systems and perceptual networks^1^. In line with this notion, auditory working memory imaging studies have revealed activations related to more general task features and to specific stimulus characteristics, respectively. For example, memory load modulated activity in fronto-parietal attention systems independent of the memorized stimulus feature^2^. On the other hand, sensory-specific activations have been found when comparing the maintenance of spatial and non-spatial information in auditory working memory with functional magnetic resonance imaging (fMRI). While the memorization of sound locations was associated with activations of posterior temporal, posterior parietal and superior frontal regions, sound identity or pattern processing involved anterior temporal and inferior frontal cortex^3^,^4^,^5^,^6^. The topography of these activations was largely compatible with the proposed dorsal and ventral streams for the processing of auditory spatial and non-spatial information, respectively^7^,^8^,^9^. In addition, some studies showed a differential involvement of left- versus right-hemispheric regions for identity versus location processing, respectively^5^,^10^. Corresponding topographic differences between auditory spatial versus non-spatial processing in working memory were also observed in studies analyzing scalp-recorded event-related potentials (ERP)^2^,^3^.

In previous magnetoencephalography (MEG) studies, we have found similar topographic differences between the processing of spatial and non-spatial sound features in working memory (see ref. 11 for a brief overview). An early study used noise stimuli convoluted with head-related transfer functions to create the impression of different levels of lateralization^12^. In the memory condition, the lateralization angle of a sample sound (S1) had to be compared with a test sound (S2) presented after a short delay period. The non-memory control condition required the detection of a possible sound intensity change in the background noise occurring instead of S2. A statistical probability mapping algorithm was applied to sensor-level spectral activity that accounted for multiple comparisons. Gamma-band activity (GBA) was enhanced at frequencies of 55–70 Hz over posterior regions during the delay phase of the memory task compared with the control condition. In addition we observed increased frontal GBA immediately prior to and during S2. Using an analogous paradigm and analysis strategy, we found enhanced GBA (65–70 Hz) over left anterior temporal/inferior frontal cortex during the maintenance of non-spatial auditory features, i.e., syllable voice onset time and formant structure^13^. In both studies, the memory conditions were also characterized by increased gamma coherence between putative sensory representation regions and frontal cortex, supporting the relevance of functional connectivity between sensory and executive networks^14^. GBA spectral amplitude and coherence during the delay phase were interpreted as reflecting the active maintenance of representations of the relevant stimulus features^15^. Subsequent work focusing on stimulus-specific activations has further supported both the role of GBA for the representation of task-relevant sound features in working memory and the distinction between the processing of spatial versus non-spatial sound features in posterior versus anterior brain regions, respectively^16^.

In these studies, we had focused our analyses on the delay phase of the working memory tasks. However, we also noted that differences between memory and control tasks might have been present already before the onset of the sample stimulus. Hints of pre-S1 activations can be seen in Figs 4e and 5 in the study by Lutzenberger *et al*.^12^ and in Fig. 4b (upper panel) in Kaiser *et al*.^13^. Here conditions were presented block-wise, i.e., participants knew which task was to be performed, and they could anticipate the onset of S1 because after the inter-trial interval, the start of a trial was signaled by a soft noise with a fixed duration of 300 ms. The aim of the present study was to directly test for activation differences between spatial versus non-spatial trials prior to S1. For this aim we presented valid pre-cues at trial onset, 2 s prior to the onset of S1. They indicated whether spatial or non-spatial stimulus features had to be processed in the ensuing auditory working memory task. If activation differences were present prior to S1, it would indicate that GBA not only represents the retention of stimulus attributes, but also the attentional orienting towards relevant information or the anticipatory establishment of task-relevant cognitive operations^17^.

The effects of pre-cues have been investigated extensively during perceptual paradigms. Predictive cues informing about the task-relevant stimuli improve performance^18^. The neurophysiological basis of this benefit is thought to consist in the modulation of sensory-selective networks by top-down influences from fronto-parietal control regions as demonstrated by fMRI^19^,^20^,^21^. Most electroencephalography (EEG) or MEG research on anticipatory signals has focused on non-phase-locked spectral activity that does not depend on a triggering event. Visual spatial attention is related to alpha-band (~8–13 Hz) desynchronization in posterior brain regions contralateral to the attended hemifield and synchronization contralateral to the unattended hemifield, putatively reflecting facilitated processing of relevant input versus inhibition of irrelevant information, respectively^22^,^23^,^24^,^25^. Corresponding alpha-band activity modulations have been suggested for auditory spatial attention^26^,^27^,^28^,^29^. Cues directing attention to auditory versus visual input typically led to parieto-occipital alpha-band activity increases, putatively reflecting a suppression of visual processing^30^,^31^. While most studies have reported effects in the alpha band, there is some evidence for increased GBA in response to informative cues in visual attention tasks^32^. In the auditory domain, Ahveninen *et al*.^33^ found increased GBA in combined MEG/EEG measurements in right inferior temporal cortex to contralateral cues in an attention task. Performance was positively correlated with GBA in brain regions including fronto-insular, auditory, and temporo-parietal cortices.

Beneficial effects of pre-cues have been observed also in working memory tasks^34^. For example, fMRI studies have shown an activation of sensory-selective regions following cues informing about the subsequently presented stimulus category^35^. This modulation as well as working memory performance was correlated with the magnitude of functional connectivity increases between sensory and fronto-parietal networks. In addition, cues predicting distraction elicited an increased activation of attention control regions, possibly reflecting the greater need for focusing on the relevant information^36^. Converging evidence was obtained in a recent EEG study investigating pre- and retro-cues in a visual working memory paradigm^37^: valid pre-cues elicited anticipatory attention shifts that were accompanied by lateralized late ERP components and alpha-band activity and led to improved performance.

In summary, anticipatory activations have been reported in visual attention tasks and some working memory studies. In EEG/MEG, modulations of pre-stimulus alpha-band power have been reported. In contrast, there is less evidence for preparatory activations in auditory cortex or for a role of GBA. The present study assessed possible activation differences between a task cue and the presentation of the sample stimulus that was to be maintained in auditory working memory. A previous analysis of the present data used linear discriminant functions to decode task-specific patterns in the MEG broadband signals^38^. Task-specific signal patterns were indeed identified during the pre-encoding period, and the same patterns recurred during later task phases, supporting the notion of the establishment of a task set preceding the presentation of the to-be-remembered stimulus. The present paper describes a re-analysis of these data with a focus on spectral activity between 5–120 Hz, including the alpha, beta and gamma ranges. Specifically, we searched for three-dimensional clusters defined by sensor space, time and frequency that differed between the auditory spatial and non-spatial working memory tasks. We expected anticipatory activation in the gamma band because we previously found task-specific effects in this frequency range during the delay phase of working memory tasks that might have been present already at task onset^11^. We hypothesized that increased pre-S1 GBA should be localized over the putative auditory dorsal versus ventral streams during the preparation for the spatial versus non-spatial auditory working memory task, respectively. In addition, we explored the alpha and beta bands because of their implication in anticipatory attention as described above.

## Results

**Figure 1:**
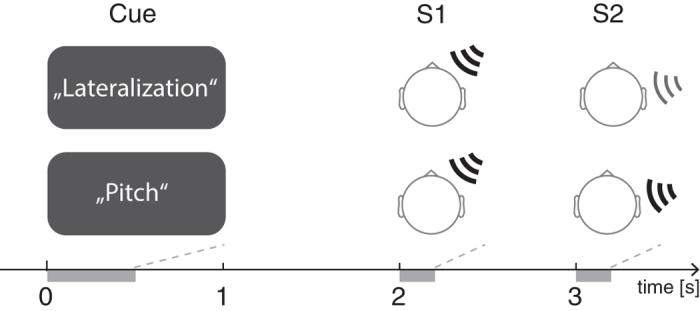
Trial structure. A visual cue (500 ms) indicated the task-relevant sound feature (lateralization or pitch). After the remaining 1.5 s of the pre-encoding phase, the sample stimulus S1 was presented for 200 ms. The test stimulus S2 followed after a 800-ms delay period. A soft midline noise was presented during both pre-encoding and delay phases.

We assessed MEG spectral activity during an auditory working memory task (Fig. 1). A valid visual cue at trial onset indicated whether pitch or lateralization was the task-relevant feature. A sample sound was presented after the 2-s pre-encoding phase. It was followed by an 800 ms delay period after which a test sound was presented. Participants had to decide whether or not the test stimulus matched the sample sound with regard to the cued feature.

### Behavioral data

The individual adaptation of task difficulty was successful in generating demanding tasks with comparable performance between conditions. The correct response rate amounted to 85.7% (SD = 6.8%) for the lateralization task and 88.1% (SD = 6.4%) for the pitch task (t(14) = 1.05, p = 0.31). Similarly, there was no difference in reaction times (lateralization: 880 ms, SD = 115 ms; pitch: 849 ms, SD = 129 ms; t(14) = 1.48, p = 0.16).

### MEG data

**Figure 2:**
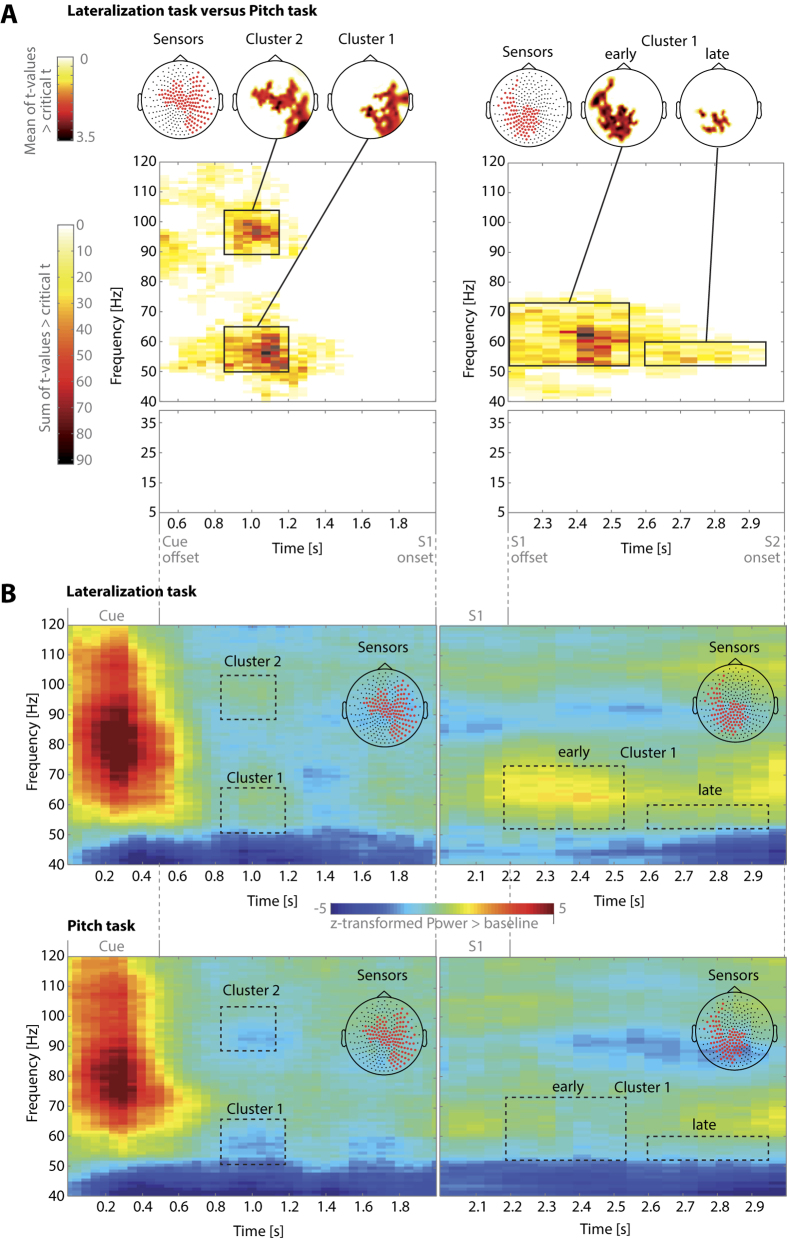
(**A**) Results of time-frequency cluster analysis comparing the lateralization with the pitch task for the pre-encoding phase between cue offset and S1 onset (left panel) and the delay phase between S1 offset and S2 onset (right panel). The top row shows MEG sensor maps (seen from above, nose up). The leftmost map indicates all sensors (marked with red asterisks) involved in any of the significant clusters; the other two maps show the topographic distributions of the mean t-values for each of the two clusters in the pre-encoding phase (left panel) or at different time ranges for the cluster during the delay phase (right panel). The time-frequency plots below depict the sum of t-values across all sensors in the cluster at each time-frequency tile. Note the compressed time scale for the pre-encoding compared with the delay phase. There were two separate clusters in the pre-encoding phase (left panel) and a single cluster in the delay phase (right panel). Both clusters showed increased activity for the lateralization compared to the pitch condition. In contrast, no significant clusters were found at frequencies below 40 Hz in either of the two task phases. (**B**) Time courses of spectral activity compared with a pre-trial baseline (−1.5 to −0.5 s prior to cue onset). Z-transformed spectral power is shown for the pre-encoding and the delay phase separately for each task. Based on the findings of the direct comparison of the tasks (part A of this figure), this analysis was restricted to the gamma frequency range (40–120 Hz) and to the sensors that constituted the significant clusters for the direct contrasts between tasks (union of clusters 1 and 2 for the pre-encoding phase, cluster 1 for the delay phase). Insets in each time-frequency plot show MEG sensor maps with the positions of included sensors. Dotted rectangles delineate the time-frequency windows with the most pronounced differences between tasks, corresponding to the solid rectangles in part A of the figure.

For the pre-encoding phase, the results of the time-frequency analysis for the comparison between the lateralization and the pitch task are shown in Fig. 2A (left panel). Cluster randomization analysis confirmed two clusters in the gamma range with higher power for the lateralization than the pitch task. One of these three-dimensional (sensor, frequency, time) clusters (p = 0.016) was centered at frequencies between approximately 50–65 Hz in the latency range of about 0.8–1.2 s after trial onset (cluster 1). Cluster 2 (p = 0.038) was centered at 90–100 Hz in a similar time range as the first. Both clusters were localized at sensors over right posterior and bilateral central sensors. There were no other significant clusters, neither in the lower frequencies nor for the reverse contrast of pitch versus lateralization condition (p > 0.15 for all clusters).

We applied the same analyses as for the pre-encoding period also to the delay phase (2.2–3 s post trial onset). The results are depicted in the right panel of Fig. 2A. There was one significant cluster (p = 0.012). Similar to the pre-encoding period, this cluster showed higher activation for the lateralization than pitch task in the gamma range. It was centered at frequencies of about 50–65 Hz and covered almost the entire delay phase from 2.2–2.9 s after trial onset. The cluster was localized at sensors over posterior, central and left temporal sensors. Again there were no effects in the lower frequencies, and there were no increased activations for the pitch task compared with the lateralization task in the delay period (p > 0.36 for all clusters).

To evaluate the findings from the direct statistical contrast of both tasks, we additionally explored the general spectral changes elicited by the experimental manipulations. Activation changes for each of the tasks relative to a pre-trial baseline are shown in Fig. 2B. Following a strong broadband gamma power increase during the presentation of the visual task cue, GBA increases at frequencies of about 50–60 and 90–100 Hz in a time window centered at about 1 s after trial onset characterized the lateralization task. In contrast, there were relative GBA decreases in roughly the same time-frequency windows for the pitch task. These activations/deactivations were clearly separable from the response to the visual cue both in terms of frequency and latency. During the delay phase, GBA increases at frequencies between about 55–75 Hz were clearly more pronounced and temporally extended for the lateralization than the pitch task.

## Discussion

Previous studies have shown topographically distinct increases of GBA over posterior versus anterior cortical regions during the delay phases of spatial versus non-spatial auditory working memory tasks, respectively^12^,^13^,^16^. To assess whether MEG spectral activity differs between both types of tasks already prior to the sample stimulus, the present study focused on the pre-encoding phase of an auditory working memory task. We used valid visual cues at trial onset that indicated whether sound frequency or perceived lateralization of a subsequently presented sample stimulus S1 had to be memorized in a given trial. Cluster randomization analysis confirmed increased GBA during the pre-encoding phase of the lateralization task compared with the pitch task. Spectral activity differences were found in two clusters at frequencies of about 50–65 and 90–100 Hz, respectively. Both activation clusters peaked about 0.3–0.7 s after cue offset, i.e. 1.2–0.8 s prior to S1 onset. In contrast to expectations, there were no relative spectral activity increases for the pitch compared with the lateralization task. The exploration of activation changes relative to baseline showed that the effects were attributable to a combination of increased GBA during the lateralization task and decreased GBA during the pitch task. Importantly, effects were restricted to the gamma band, whereas there were no differences in frequencies below 40 Hz. The topography of GBA increases for the lateralization task over right-hemispheric parieto-occipital and bilateral central areas is partly consistent with an involvement of auditory dorsal stream regions in the processing of spatial sound features in working memory^2^,^3^. The right lateralization of the posterior GBA is compatible with the proposed right-hemispheric dominance for attention or memory processing of spatial information^5^,^10^,^39^,^40^. However, as no source analysis was performed, the attribution of the observed effects to cortical sources has to remain tentative.

The new finding of the present study consists in the presence of task-related differences in GBA *prior to* the presentation of the memorandum. Task-specific anticipatory activations have been reported in human EEG since the seminal work on the motor readiness potential^41^ and on negative slow potential shifts during the preparation for perceptual and cognitive tasks^42^. While these early studies have mainly revealed hemispheric differences in task involvement, a recent MEG study reported differences in preparatory broadband source activity in the frontal eye fields and superior temporal sulcus during auditory spatial versus pitch attention tasks, respectively^43^. fMRI studies in the visual domain have reported preparatory activations during various paradigms including attention tasks^21^,^44^, visual working memory^45^ and task switching paradigms^46^. For example, examining auditory attention control with a catch-trial design in fMRI revealed partly overlapping networks with stronger activation in left inferior frontal cortex for pitch cues and in precentral sulcus and superior parietal lobule for spatial cues^47^. These investigations typically found preparatory activations in regions that were also involved in subsequent task processing. The present pre-encoding GBA increase was not sustained until the onset of S1. It might therefore have reflected the initial establishment of a task set rather than the attentional preparation for stimulus processing. As the task switched frequently between trials, the observed effect might also reflect the reorientation of the attentional focus on sound lateralization. While sustained task-set activity has been observed in single-cell recordings, in human brain imaging task-set information manifested itself either as a signal pattern across multiple voxels within a region or as a pattern of interactions between brain regions^17^. The method of the present study might therefore not have been able to identify the neural correlates of sustained task-set activity.

GBA increases for the lateralization task were found also across most of the delay phase. The comparison of task-related activations with baseline suggested that this effect was mainly accounted for by stronger GBA increases in the lateralization than pitch task. On the one hand, this activation cluster was highly similar to the 50–65 Hz pre-encoding cluster in terms of frequency range. On the other hand, it showed a partly different sensor-level topography with a stronger involvement of sensors over the left hemisphere and over temporal in addition to posterior-central regions, possibly indicating independent cortical sources of the pre-encoding and delay-related clusters. Moreover, the 90–100 Hz pre-encoding cluster was not observed in the delay phase. These findings argue against a preparatory activation of the same networks that were involved in the actual working memory processing of the sample stimulus. However, a perfect correspondence between pre- and post-encoding activations was not to be expected, given that different processes are relevant in both periods. While the establishment of a task set or an attentional orientation towards spatial sound processing might have characterized the pre-encoding period, the delay phase involved perceptual processing and maintenance of the actual S1 stimulus and, potentially, the preparation both for the processing of S2 and the comparison of both sounds. Given that sounds were presented in the right hemifield, the left-hemispheric predominance of activity during the delay phase could indeed reflect a stronger involvement of stimulus processing.

Based on our previous work on GBA during spatial versus non-spatial auditory working memory^12^,^13^,^16^, we had expected to find relative enhancements of spectral activity with distinct topographies both for the lateralization and the pitch tasks. However, we found no pitch task-specific GBA increases neither for the pre-encoding period nor the delay phase. We can only speculate about the reasons for this discrepancy. The lateralization task appeared to be slightly more difficult than the pitch task. However, this difference was not statistically significant, making it unlikely that the relative GBA increase for the lateralization task is attributable to a higher demand for processing resources. The lack of pitch task-related GBA in the delay phase was in contrast to our above-cited previous studies. However, there are a number of significant differences between the previous and the present study, making a comparison difficult. In our earlier working memory studies, we did not compare spatial and non-spatial memory tasks directly but contrasted each memory task with a non-memory control task^12^,^13^. In addition, the earlier studies used a different analysis procedure. Instead of cluster randomization analysis, a statistical probability mapping of spectral amplitude values averaged across most of the delay phase served to identify combinations of individual sensors and (narrow) frequency ranges with the most pronounced differences between conditions. The more recent study^16^ is even less comparable to the present one, because it followed a very different rationale, aiming to detect GBA differences between individual stimulus features during spatial and non-spatial tasks instead of differences between tasks.

At least two other investigations have also reported a predominance of cortical activation in preparation to spatial compared with non-spatial processing. In a visual attention study, verbal cues that indicated either the to-be-attended hemifield or color both elicited fMRI activation of the fronto-parietal attention control network^44^. The direct contrast between conditions, however, showed more pronounced responses to spatial cues in superior frontal and parietal cortex, whereas there were no relative enhancements for the non-spatial cues. The fronto-parietal network’s preference for spatial attention control was tentatively attributed to the existence of spatially selective regions within this network^48^. However, this explanation does not hold for the present findings where the cue indicated the task-relevant feature instead of a particular position in space. In the auditory domain, an fMRI study compared selective attention to sound location versus pitch by instructing participants to detect infrequent short-duration sounds while attending to either high- or low-pitch harmonic sounds presented to one ear (non-spatial task) or to either left- or right-ear sounds of constant pitch (spatial task)^49^. They found stronger activations in the spatial than non-spatial task in bilateral premotor/supplementary motor cortex, left posterior temporal and right inferior parietal regions. Similar to the present study, there were no activations specifically related to attention to pitch. The authors speculated that more complex stimuli or tasks might be needed to activate sound pattern or object processing regions. Some of the fMRI studies reporting separate activations for spatial versus non-spatial processing have indeed used either more complex stimuli like environmental sounds^6^,^50^ or required more demanding processing of either sound feature^3^. This explanation could potentially be applied also to the present study using abstract RIN sounds.

Alternatively, there might be a generally more dominant role of spatial attention compared with attention towards features defining the identity of an object. At the behavioral level, location appears to be the more salient feature for both visual and auditory object processing^51^,^52^. For example, performance in an auditory retro-cueing task benefitted more from spatial than semantic attention cues^53^. At the neural level, according to the dual-stream model^7^ the auditory and visual dorsal streams include similar regions, whereas there is a clear segregation of the ventral streams in both modalities. The possible supramodal nature of the spatial processing system is supported by evidence from both fMRI and event-related potentials suggesting that auditory and visual spatial attention rely on at least a partly shared network^26^,^54^,^55^. Thus the preparation for spatial processing likely relies on a supramodal cortical network for spatial attention that might be topographically more extended, and thus more ‘visible’ in the present MEG analysis, than the network specialized for attention to pitch.

Our exploration of frequencies across the range of 5–120 Hz found no differences between the spatial and non-spatial conditions in frequencies below 40 Hz. This was true for both pre-encoding and delay periods. Modulations in the alpha range are typically observed in spatial attention paradigms. Here contralateral alpha desynchronization and ipsilateral synchronization are well established in the visual and somatosensory domains^22^,^56^, and similar evidence has emerged recently also for auditory spatial attention tasks^26^,^27^,^28^,^29^. In contrast, the pre-cues in the present study did not direct spatial attention to certain locations, but indicated whether sound lateralization or pitch was the task-relevant stimulus feature. The right-hemispheric predominance of GBA increases *ipsilateral* to the subsequent sample sounds also speaks against an interpretation of the observed activations as reflecting spatial attention. Therefore, the lack of lower-frequency effects in the pre-encoding phase of our study is not surprising. The selection or preparatory activation of an attentional set thus seems to be related to activity in the gamma-band range. Concerning the delay phase, in previous auditory working memory studies we found alpha-band activity increases when comparing memory maintenance with baseline^57^, and positive correlations between alpha and beta activity with memory load^58^, but no differences between the spatial and non-spatial conditions during stimulus maintenance^12^,^13^. Therefore we had not expected lower-frequency effects in the present investigation.

## Conclusion

The present auditory working memory study revealed task-related pre-encoding MEG activity in the gamma-band at frequencies of 50–65 and 90–100 Hz localized over predominantly right posterior and bilateral central cortex. GBA at 50–65 Hz was increased also during the maintenance phase of the spatial working memory task. However, the different sensor-level topography suggested separate cortical generators of pre-encoding versus delay-related activations. While GBA in the pre-encoding period reflected the preparatory activation of cortical networks involved in auditory spatial processing, activity during the delay phase was related to the perceptual and memory processing of lateralized sounds in working memory. No task-related modulations were observed in frequencies below 40 Hz, suggesting that alpha-band activity is not involved in task set selection. There were no relative increases of spectral activity for the non-spatial condition in either task period, which could be attributable to the relatively low complexity or salience of the employed sounds or to the general dominance and larger cortical extent of networks involved in the control of spatial attention. The present findings suggest that, in contrast to the notion that GBA plays a role mainly for sensory-driven processing^56^,^59^, signals in this frequency range are associated with top-down-driven processes such as preparatory activation of task-relevant networks.

## Methods

### Participants

Twenty healthy, right-handed adults took part in the study (11 females, mean age: 24.3 years, age range: 20–29 years). All of them reported that they had normal hearing abilities and no diseases of the auditory system. Five subjects who did not achieve correct response rates above 70% in at least 7 out of 10 recording blocks (see section ‘Procedure’ below for details) were excluded from the analyses. This left 15 participants (8 females, mean age: 24.5 years, age range: 20–29 years). All subjects gave their written and informed consent. They received a remuneration of € 10/hour. The ethics committee of the Goethe University Medical Faculty approved the study. The methods were carried out in accordance with the relevant guidelines and regulations. Informed consent was obtained from all subjects.

### Procedure

Participants performed an auditory working memory task in which the relevant stimulus feature (pitch or lateralization) was indicated by a visual cue at the beginning of each trial. Subjects were seated upright in a magnetically shielded room (VAC, Hanau, Germany). The trial structure is shown in Fig. 1. A trial started with a 2-s pre-encoding phase during which a soft midline white noise was presented binaurally (intensity: 64 dB (SPL)). During the first 500 ms of the trial, a visual cue informed about which stimulus feature was to be remembered. The cue was a word presented presented at the center of a backprojection screen placed about 50 cm in front of the subject (German translations of direction (“Richtung”), pitch (“Tonhöhe”), both (“Beides”) or pause (“Pause”)). The pre-encoding phase was followed by the sample stimulus S1 (duration: 200 ms, intensity: 84 dB (SPL)), see section on “Stimuli” below. During the following 800-ms delay phase the softer white noise was presented again. The delay phase was followed by a second 200-ms test stimulus S2 (84 dB (SPL)). This was followed by a 1500-ms response phase during which participants had to judge whether S1 and S2 matched or differed with respect to the cued sound feature. The response (match or non-match) had to be given as fast as possible within a 1500-ms response period by pressing a button on a fiberoptic response pad triggering a light barrier (LUMItouch; Photon Control, Burnaby, Canada) with the right or left index finger (response sides were counter-balanced across subjects). The duration of the subsequent inter-trial interval varied randomly between 1550 and 2300 ms in steps of 50 ms. The sequence of events was controlled by Presentation (Neurobehavioral Systems). Subjects were instructed to sit still and keep their eyes open, looking at a fixation cross on a screen in the center of their visual field about 2 m in front of them. They were asked to try to blink only during the inter-trial intervals.

The following four tasks were presented in a pseudo-randomized order. In the lateralization task participants had to judge whether S1 and S2 matched in terms of perceived lateralization, in the pitch task they had to compare their pitch. In both tasks, the task-irrelevant stimulus feature varied randomly between S1 and S2, i.e., participants had to focus their attention on the task-relevant feature. There were two additional conditions: a task where participants had to judge whether sample and test stimulus matched on both lateralization and pitch, and a passive control task during which subjects were not required to perform a working memory task but only to respond as fast as possible with the left or right index finger to S2. However, these conditions were not included in the present analyses because they did not allow discriminating between pitch and lateralization processing.

Prior to the recordings participants performed at least 16 practice trials until they reached a minimum performance of 70% correct for each condition. Visual feedback was given after each trial. Each participant then performed 10 runs (duration: 6.5 min) consisting of 64 trials each (16 trials per condition). Participants received visual feedback about their correct response rate after each run.

### Stimuli

Regular-interval noise (RIN) sounds with different pitch and lateralization were created with custom Matlab (MathWorks R2010a) scripts adapted from Griffiths *et al*.^60^. Different pitch values were obtained by repeatedly (16 iterations) adding a time-shifted copy of low-pass filtered white noise (cutoff: 1600 Hz). A constant delay d with every summation yields a perceived pitch with a frequency of 1/d (given 1/d > 30 Hz). The RIN stimuli were additionally multiplied by a 20-Hz sinusoidal envelope curve and fade-in (fade-out) cosine amplitude modulation of 50 ms at the beginning (end) of the stimulus. Presenting the binaural stimuli with an interaural time delay (ITD) created the impression of sound lateralization. All sounds were presented earlier to the right than to the left ear, giving the impression of right-lateralized stimuli. Stimuli were generated with a sampling rate of 44.1 kHz and had a duration of 200 ms. The intensity of the sounds measured with a Reed 120–0014 sound level meter (TechniCal Systems Inc., Hamilton, Canada) amounted to 84 dB (SPL) at the basic frequency of 220 Hz and 200 μs ITD. Auditory stimuli were presented binaurally via air-conducting tubes with ear inserts (E-A-Rtone 3A, Aearo Corporation, Indianapolis, USA).

Sample and test stimuli were obtained by combinations of two pitch (low: 220 Hz, high: 300 Hz) and two ITD (medial: 200 μs, lateral: 600 μs) values. To obtain comparable performance levels between conditions and across subjects, a new set of these four stimuli was generated for every run by reducing (or increasing) the higher value on the two stimulus dimensions while keeping the lower values constant (pitch: 220 Hz, ITD: 200 μs). Thus, when performance in the previous run was below 70% or above 90%, the high pitch and the right lateralization values were decreased or increased to make the pool of four stimuli more similar or dissimilar, respectively, and the delayed match-to-sample task thereby harder/easier for the subsequent run. This procedure resulted in a mean high pitch of 267.0 Hz (SD: 24.5) and a mean lateral ITD of 546.2 (SD: 52.4).

### Data recording

MEG was recorded using a whole-head system (CTF-MEG, VSM MedTech Inc., Coquitlam, Canada) comprising 275 magnetic gradiometers with an average distance between sensors of about 2.2 cm. Signals of four defunct channels were discarded. The signals were recorded continuously at a sampling rate of 1200 Hz with an anti-aliasing filter at 300 Hz. The final signal was computed using a synthetic third-order gradiometer configuration to suppress environmental noise and downsampled at 300 Hz. Each subject’s head position was determined with localization coils fixed at the nasion and the preauricular points and monitored throughout each recording block to ensure that head movements did not exceed 0.5 cm at any time. To control for eye- and heart-related artifacts, we recorded both the electrooculogram from four electrodes above and below one eye and lateral to both eyes and the electrocardiogram from two electrodes placed on the clavicles.

### Data analysis

Data analysis was performed using Matlab (The MathWorks R2010a and 2012b) and the matlab-based Fieldtrip Toolbox (ref. 61, http://fieldtrip.fcdonders.nl). Signals were bandpass-filtered between 0.1–150 Hz and notch-filtered at 48–52 Hz to remove electric line noise. The data were cut into epochs of 5.5 s duration, including 1.5 s before and 4 s after trial onset.

#### Artifact correction

Artifact correction was performed with a semi-automatic Fieldtrip routine. To detect muscle artifacts, the signals were bandpass-filtered at 110–140 Hz. After z-transformation, epochs were excluded from further analysis if z-values exceeded a threshold value of 12. For sensor jumps, unfiltered epochs containing data exceeding a z-value of 30 were eliminated. Cardiac and ocular artifacts were corrected using independent component analysis (ICA). Independent components were identified whose correlation with the electrooculogram or electrocardiogram channels exceeded the mean correlation coefficient of all independent components with these channels by more than 3 standard deviations. Independent components reflecting artifacts were removed before back projecting the signal into sensor space. As rejection of artifactual epochs could lead to unequal numbers of trials per condition, the number of epochs entering the analyses was reduced to match the condition with the smallest number of artifact-free epochs for each participant. This was achieved by randomly rejecting the number of excess epochs. After artifact correction, on average 133.3 (SD = 17.4) trials per participant and condition remained for analysis.

#### Spectral analysis of MEG data

Spectral analysis was performed on single-trial basis to obtain estimates of total, non-phase-locked or “induced” activity. Previous studies have indicated anticipatory activations in the alpha and gamma bands^27^,^30^,^32^. Here we explored frequencies in the full range 5–120 Hz (including alpha and gamma bands) to identify any potential differences between conditions in spectral activity during the pre-encoding as well as during the delay phase of the task. We used separate strategies for the analysis of low- and high-frequency activity. For the lower frequency range (5–40 Hz), we applied a sliding window of 800 ms in 50-ms steps and a single taper (Hanning window), giving a frequency resolution of 1.25 Hz. For higher frequencies (40–120 Hz), we used a 400-ms time window sliding in 50-ms steps. We applied 5 tapers, leading to ±7.5 Hz smoothing. For both low and high frequencies, Fast Fourier Transforms were used to calculate the frequency spectrum in steps of 1 Hz. Square roots of power values were computed to obtain more normally distributed spectral amplitude values.

We performed a cluster randomization analysis as implemented in Fieldtrip^61^ to compare pre-encoding spectral activity between both tasks. As we had no a-priori assumptions about topography, frequency or time range, the analysis was performed for all 271 intact sensors and a wide time window from 500 ms through 2000 ms post trial onset (i.e., spanning the entire pre-encoding phase except for the first 500 ms to reduce contamination by responses to the visual cue). Separate analyses were conducted for the lower frequencies (5–40 Hz) and gamma (40–120 Hz). First, repeated-measures t-tests with an uncorrected significance criterion of p < 0.05 were performed for each sensor and each time-frequency bin. Three-dimensional clusters were formed including sensors with significant time-frequency bins neighboring either in space, time or frequency. Then a summed t-value was calculated for each cluster across all significant bins in those three dimensions. Second, the significance of the clusters was tested with a permutation test procedure correcting for multiple comparisons. To obtain a t-value distribution under the null hypothesis, condition data was reassigned to either lateralization or pitch task by randomly flipping the sign of the contrast between tasks at the level of the participant. For each of 5000 permutations randomly drawn out of the possible 2^n^ permutations, the summed t-value of the largest cluster was determined. The observed clusters were considered significant if their summed t-value ranged among the top 5% of maximum t-values in the distribution obtained by the permutation procedure. The same approach was subsequently applied to spectral activity differences between tasks in the delay phase (2.2–3 s post trial-onset) to explore the relationship between pre-encoding and delay-related activations.

For a better evaluation of the difference effects obtained by the cluster randomization analysis, we additionally assessed spectral changes separately for both experimental conditions and task phases compared with the average across a pre-trial baseline of −1.5 to −0.5 s prior to the onset of the visual task cue. Z-transformed spectral power changes were calculated separately for the pre-encoding and delay phase. This analysis was restricted to the frequency range where the statistical comparison between tasks had shown significant effects, i.e., the gamma frequency range between 40–120 Hz. Also, we included only those sensors that constituted the significant clusters obtained by the direct statistical contrast between tasks, i.e. different sensors were included in the analysis for the pre-encoding than for the delay phase.

## Additional Information

**How to cite this article:** Kaiser, J. *et al*. Pre-encoding gamma-band activity during auditory working memory. *Sci. Rep.* **7**, 42599; doi: 10.1038/srep42599 (2017).

**Publisher's note:** Springer Nature remains neutral with regard to jurisdictional claims in published maps and institutional affiliations.
